# Therapeutic work to enhance parental mentalizing for parents with ACEs to support their children's mental health: A theoretical and clinical review

**DOI:** 10.3389/frcha.2023.1094206

**Published:** 2023-03-16

**Authors:** Daphna G. Dollberg, Keren Hanetz-Gamliel

**Affiliations:** School of Behavioral Sciences, Academic College of Tel Aviv-Yaffo, Tel Aviv, Israel

**Keywords:** parental mentalization, ACES, parent psychotherapy, parental reflective functioning, intergenerational trauma

## Abstract

This review outlines the literature concerning the impact of adverse childhood experiences (ACEs) on parenting, focusing on how childhood trauma in parents might impede the development of adaptive parental mentalizing skills. Non-adaptive parental mentalizing may lead to non-mentalizing cycles between parents and children, which can put the child's mental health at risk. When parents who have endured ACEs have to cope with their children's mental health problems, they may have to deal with a double dose of parental stress related to their own traumatic history and their children's emotional difficulties. This heightened parental stress may further shake the parents' mentalizing skills. In line with this special issue's topic, we propose the promoting and restoring of parental mentalizing as a treatment goal for parents who have endured ACEs and whose children face mental health difficulties. We review the empirical and clinical evidence regarding the benefits of effective parental mentalizing and the availability of techniques to enhance it. We argue that working therapeutically and focusing on supporting and advancing parental mentalizing is an effective and feasible treatment goal with parents who endured ACEs. We demonstrate how we use these interventions through fictional vignettes from our therapeutic work and offer recommendations for clinical work with parents with traumatic histories.

## Introduction

Accumulating evidence indicates that parents' adverse childhood experiences (ACEs), consisting of abuse, maltreatment, neglect and living in a dysfunctional household before the age of 18, can interfere with parenting (1–3). Parents' ACEs may also impede their children's development, increasing the risk of the latter developing insecure attachment and behavior problems (4–7) and encountering future trauma (8). The terms “intergenerational transmission of trauma” (4, 9, 10) and “cycles of maltreatment” (11) have been used to highlight the cyclical and transgenerational nature of trauma within families. However, there is evidence indicating that not all parents who suffered childhood adversity have problems parenting [see (8, 12) for a review]. Furthermore, the impact of parents' ACEs on their children's developmental outcomes varies (9).

In light of the attachment and mentalization theories, we explore how exposure to ACEs interrupts the development of a secure attachment in the parent and may create a tendency to affect dysregulation, which consequently may interfere with the development and maintenance of adequate parental mentalizing skills. Inadequate parental mentalizing skills may interfere with the parent's ability to accurately read and infer the child's mental needs, leading to insensitive and ineffective parenting, which, in turn, may put the child's development and wellbeing at risk. We propose that using mentalization theory and mentalization-based techniques can help enhance and restore the mentalizing skills of parents who have suffered ACEs. These skills can serve as a protective factor for the parent, the child and the parent-child relationship. Methods for improving these skills include using the therapist's mentalizing stance, identifying, modulating and exploring parents' arousal and consequent mentalization collapses, and helping parents reflect on their own mental processes linked with their traumatic childhood experiences.

### Childhood adversity and parenting

Building on the first large-scale ACE study in the US by Felitti and his colleagues (13), research has shown that growing up in a dysfunctional household and/or being exposed to maltreatment, abuse or domestic violence increases one's risk of poorer physical health outcomes (13–15) and a host of mental health and socioemotional difficulties (1, 15–17). A recent meta-analysis looking at ACEs internationally reported that they are common in clinical patients as well as low-risk populations and that their rates are underestimated (18). Hoppen and Chalder (19) proposed a theoretical model according to which exposure to ACEs early in life may lead to the dysregulation of the autonomic nervous system and hyper-reactivity of the hypothalamic-pituitary-adrenal axis that interfere with the operation of the stress response. A recent meta-analysis confirmed that exposure to ACEs is associated with dysregulation of the stress response system, leading to blunted cardiovascular and cortisol stress reactivity in lab-administered stress tasks (20).

Childhood trauma, especially when inflicted by the attachment figure, involves the failure of the latter to respond sensitively to the child or to respond in a way that increases the child's distress. Such responses undermine the child's development of organized strategies to regain and achieve affect regulation. Consequently, childhood trauma increases the risk of a child developing an insecure attachment style, a low threshold for frustration, and a reliance on compromised and defensive affect regulation strategies (21, 22). Childhood trauma is also associated with ineffective mentalizing. An environment of abuse and maltreatment is often one where interest in the mental state of others is lacking or the child's intentions and wishes are misinterpreted and distorted (8, 23). Thus, facing repeated distorted attributions of their own intentions and those of others, traumatized children may struggle to make sense of own internal states, often experiencing confusion and difficulty in interpersonal relationships (24).

Moreover, early attachment experiences and accurate reading and inferring of the child's internal experiences set the stage for the child's epistemic trust. Such trust involves the child's readiness to receive personally relevant knowledge about the social world, which is linked with effective, rapid and reliable social learning. On the other hand, the attachment figure's failure to accurately read and meet the child's attachment needs and the latter's insecure attachment, all associated with traumatic environments early in life, are likely to lead to epistemic mistrust and hypervigilance, reflected in the child's learning that one cannot rely on others (25).

Parents who experienced childhood trauma may find that their tendency toward emotional dysregulation, epistemic mistrust and hypervigilance, the likelihood of their having an insecure attachment style, and their difficulty with mentalizing put their parenting at risk (26). The emotional dysregulation may lead to an activation of the domain-specific parenting stress response system, resulting in high levels of parental stress (27, 28). Parents who have a dysregulated stress response system often find dealing with common parenting challenges such as an infant's crying, setting limits, and dealing with the child's dysregulation more difficult, making caregiving less rewarding for them (29) and further shaking their compromised coping abilities (10). Translating polyvagal theory (30) to parenting in the context of ACEs, Suardi and colleagues (10) argued that when parents who have experienced previous trauma are faced with the stress of caring for a distressed child, they automatically link it with danger and threats. This response activates their self-defense strategies with regard to their own attachment needs and inhibits the neural circuits related to communication and bonding. Absorbed in regulating their own arousal, they are less likely to attend to the child, adequately read and infer the child's bids for attachment, and provide sensitive and attuned parenting (10). In line with this theoretical formulation, as well as the finding regarding blunted stress responses following ACEs (20), evidence shows links between mothers' ACEs and decreased parental sensitivity (31), reduced emotional availability (32), and distorted attributions regarding the child's intentions and mental states (33), all of which increase the risk of insecure attachment in the child (34).

So far, the majority of research on ACEs has focused on mothers, with only limited evidence as to how fathers' adverse childhood experiences are linked with parenting. A large, US-based national sample (35) recently documented negative associations between fathers' ACEs and positive parenting behaviors such as warmth and emotional support as well as positive father-child relationships. Fathers with multiple ACEs also tended to use harsher discipline and engaged in more negative parenting behaviors. The authors concluded that, as with mothers, ACEs can create emotional dysregulation in fathers, which may lead to less positive parenting (35).

To summarize, it appears that a parent, mother or father, who grew up in a traumatizing environment, may face emotional dysregulation in many areas, including parenting. Their adverse childhood may impede the development of adequate coping resources such as secure attachment, effective self-regulating skills and adequate mentalizing capacities. The lack of these skills creates chronic struggles for the parent that may compete with the resources needed for sensitive caregiving. The lack of these skills may increase the risk of affect and behavior dysregulation and mental health difficulties in the child, eventually leading to the child's referral for mental health treatment (36). Therefore, working with children to enhance their wellbeing calls for work with the parents to improve their understanding of the child's emotional needs, enhance the parent's self-regulation and increase effective parenting (37).

### Parental mentalization

Parental mentalization refers to the parent's tendency to view the child as a psychological agent with a mind that is separate from the parent's mind (38, 39). Parental mentalizing is the capacity by which the parent can envision the thoughts and feelings (i.e., mental states) that underlie their own behavior and that of their children (40, 41). Mentalization is thought of as an umbrella concept that overlaps with a number of other important constructs (22, 42, 43). In the mentalization-based literature, mentalizing and reflective functioning are often used interchangeably (8), with reflective functioning referring to the intentional, effortful pattern of mentalization (42). While sharing commonalities with “theory of mind” and “mind reading” on one hand and empathy and mindfulness on the other, scholars of mentalization argue that it is a broader construct that involves the capacity to infer mental states, both emotional and cognitive, in oneself as well as in others (21).

Mentalizing is organized around four dimensions or poles. Mentalizing can be automatic and reflexive or controlled, intentional and reflective; it can focus on the self or on others; it moves between focusing on external, behavioral or internal, mental features of the self and others; and it combines and integrates cognition and affect (42). Adaptive parental mentalizing involves a balanced, flexible, context-dependent move along the four dimensions and between the poles. Non-adaptive parental mentalizing is characterized by a rigid fixation on one or more poles and is clustered around three typical modes. The first is the teleological mode, where the parent is fixated on external, behavioral reality and where physical action is considered the only option for modifying the child's behavior and mental state. The second is the psychic equivalent mode, a mind-state where the distinction between external and internal reality and between the child's and the parent's mind is blurred and the thinking is mostly emotional and self-centered. The third is the pretend mode, where the mental world is decoupled from external reality and given priority over it (43).

Empirical evidence shows that adaptive parental mentalization is a parental asset. It is associated with healthy socio-emotional and socio-cognitive development in children (44) and reduced risk of children's internalizing and externalizing behaviors (45, 46). Parents' mentalizing abilities help them maintain their behavioral and emotional regulation in the face of the high level of arousal related to an infant's cry (47), the child's frustration (48), and parental stress in the context of premature births (49), thus support parenting (41). Conversely, mothers who score low on parental mentalization and reflective functioning tend to exhibit disrupted parenting, and have infants with disorganized attachment issues (50) and children with mental health difficulties (51).

The benefits of a high level of parental mentalization go beyond those for the parent. Parental mentalization serves as a buffer against caregivers' mental health difficulties (52) and helps promote children's mentalizing and self-regulation skills (48). One study we conducted showed that maternal mentalization is a protective factor that attenuates the links between parents' anxiety and children's externalizing behaviors (53). In addition, we recently reported that when mothers' mentalization skills are stronger, the links between their ACEs and their children's behavior problems are weaker than those with mothers who have poorer mentalization skills (54).

### Parental mentalizing mitigating the intergenerational transmission of trauma

As noted above, childhood maltreatment and trauma are significant risks to the ability to develop one's mentalizing capacity fully. In the context of childhood maltreatment, both the parent's and the child's mentalizing capacities are likely to be impaired, contributing to the intergenerational transmission of poor mentalizing skills in families (55). However, it is important to note that childhood trauma does not always go hand in hand with ruptured parental mentalizing. In fact, the evidence points to a “loose coupling” (42) between trauma, insecure attachment and fragile mentalizing. Fonagy and colleagues (56) underscored this point. They showed that some prospective parents, despite their own childhood adversity, demonstrated a high level of mentalizing, reflecting openly and coherently on their attachment figures' harmful parenting. Importantly, these parents were less likely to have insecurely attached infants. The authors suggested that when prospective parents were able to reflect coherently and non-defensively on their painful early experiences, meaning they showed better mentalization of their childhood trauma, they were more likely to accurately infer the child's inner states and be aware of how the child saw their behavior. This ability helped the parents recalibrate their negative thoughts and feelings, and fostered benevolent parenting and co-regulation of the child, leading to the child's secure attachment (12, 42). In support of this argument, Ensink and her colleagues (8) reported that a higher level of mentalization regarding early trauma among women with histories of childhood abuse and neglect predicted less difficulty engaging in and more positive affects towards the pregnancy and motherhood. Recently, Borelli and her team (12) found that maternal mentalizing regarding early trauma among women with a history of childhood sexual abuse was associated with less likelihood of exposing their own children to sexual abuse. The accumulating empirical evidence regarding the importance of mentalization in breaking the intergenerational transmission of trauma led us to propose that enhancing and supporting parental mentalization is an important treatment goal with parents who have endured ACEs.

The importance of working through a parent's childhood trauma in order to foster sensitive parenting is not new. In their seminal paper, “Ghosts in the Nursery,” Fraiberg and her team (57) argued that an intensive, psychoanalytically oriented treatment with parents in the presence of the infant allows traumatized parents to reconnect to previously repressed and dissociated feelings of helplessness, fear and anger, freeing them to sensitively attend to the infant's distress.

However, when it comes to parents of older children who are referred to child and adolescent mental health services, the parents are not always willing to discuss their own childhood adversity in the context of their children's psychotherapy. Moreover, in typical child and adolescent mental health settings, the parents are not the identified patients. Therefore, a full exploration and working through of their childhood trauma is usually not feasible. Thus, we suggest that a more focused and less ambitious treatment goal with these parents is needed. We argue that enhancing parental mentalizing is a relevant, achievable and effective treatment goal with parents who are survivors of early childhood trauma. We suggest that the treatment focus on helping parents mentalize on how their ACEs may at times influence their parenting (parental self-reflection) and how this may impact their child (parental child-focused reflection). In the next section we elaborate on this idea.

### Therapeutic interventions to enhance parental mentalizing

Parental mentalization-based interventions [e.g., (22, 58–61)] are anchored in contemporary attachment theory and developmental research [see (22, 24, 39, 41, 60)]. These interventions are integrative in nature, drawing on psychodynamic understanding coupled with a developmentally informed, structured approach. What distinguishes mentalization-based interventions from other parental psychotherapy approaches is their distinct emphasis on promoting the parent's mentalizing and reflective functioning as a primary treatment goal (62).

Several interventions have been designed to promote parental mentalizing, mostly among mothers of infants and toddlers. Minding the baby (63), MBT-P (64) and the Mother and Toddler Program (65) are examples of interventions that specifically target the mother's mentalizing as their treatment goal [see (51, 66) for a review]. Empirical evidence shows the benefits of these interventions [see (66)], particularly in promoting the development of secure attachment (63) and reducing behavior problems in children (61, 67, 68). While showing promising effectiveness, these interventions have several limitations. They are focused primarily on mothers of infants and toddlers and work within settings that are relevant for these populations such as home visits and/or group work. Mentalization-based interventions for mothers and fathers of older children are rare [see (22, 69) as exceptions]. Furthermore, a systematic evaluation of mentalization-based interventions for older children and in the context of parental therapy in conjunction with their children's therapy is lacking (62). The goal of our review and clinical discussion is to argue for and demonstrate clinical implementation of mentalization-based interventions with parents who endured ACEs in child mental health settings. Our review sums empirical evidence regarding the benefits of parental mentalization for parents and children in general and for those who endured childhood adversity in particular. Furthermore, compelling evidence shows the effectiveness of parental mentalizing-focused interventions for parenting infants and toddlers. We argue, based on the evidence reviewed, that setting parental mentalizing as a treatment goal and using mentalization theory and techniques to guide the treatment may be useful for parents of older children and in conjunction with individual therapy for the child.

### Using mentalization theory and techniques with parents who have experienced ACEs

In line with this special issue's topic, which focuses on ACEs and children's mental health, and the recent call for using ACE-informed interventions with parents, we describe how we use mentalization theory and techniques to enhance and restore parental mentalization skills among previously traumatized parents to help support their children's mental health.

The transactional developmental model (70) argues that parents and children affect each other over time through mutual representations and interactions. Based on the literature reviewed above, we maintain that traumatized parents whose children experience mental health problems are likely to experience a double dose of stress. Aside from their own trauma, their children's mental health difficulties are likely to lead to additional stress, which together may shake their mentalizing abilities and increase the risk of repeated non-mentalizing and exacerbation of the child's mental health and behavioral difficulties. On the other hand, the parent's capacity to look beyond the child's difficult behavior to the child's subjective experience may help the parent maintain self-regulation so that the parent does not respond in ways that escalate the child's distress and oppositional behavior (71).

In general, enhancing and supporting parental mentalizing is thought to help parents in three main areas. First, it helps them develop or regain their ability to look past the child's behavior and to be curious about the child's inner experiences. Second, it promotes parents' awareness of their own affects and behavior, especially in conflict with the child, when they may lose their affect regulation and mentalizing abilities. Third, parental mentalizing encourages parent-child interactions in which the child feels secure and understood. Such an environment promotes the child's self-regulation, mentalizing and trust in the adult world (22). These goals are especially important when working with parents who endured ACEs. These parents are likely to lose their mentalizing stance vis-a-vis the child when aroused by the child's behavioral and affect dysregulation or when facing a conflict with the child.

Working therapeutically to enhance parental mentalization consists of collaborative discussions of the possible mental states that underlie the parent-child interactions and observed behaviors. The therapist provides scaffolding to support the parents' mentalizing. If the parents insist on a narrow, behaviorally focused and/or developmentally improbable explanation for the child's behavior, the therapist can give voice to the child's imagined and/or hypothetical experiences (60). Central to mentalization-based practice is the therapist's mentalizing stance, meaning presenting himself or herself as curious, inquisitive and in a “not knowing” position. The therapist's mentalizing stance also involves paying attention to moments when his/her own ability to mentalize either the child's or the parent's inner experiences is lost, which may happen during challenging in-session exchanges (62).

We believe that mentalization theory and the techniques it offers are especially useful when working with traumatized parents because of the attention they pay to several issues that are pivotal to working with survivors of childhood trauma: establishing epistemic trust and a therapeutic alliance; regulating the parents' emotional arousal; following the parents' lead; and handling the parents' mentalizing collapses and non-mentalizing modes. Our clinical discussion is accompanied by vignettes that demonstrate our therapeutic work. The interventions described throughout the vignettes are drawn from our clinical work. However, in order to protect our patients' privacy, they are fictional, blending together several cases. We use them to demonstrate a therapeutic principle rather than discuss a specific case in depth. In each vignette we also included our thinking in parentheses regarding how it demonstrates a specific mentalizing-related concept and/or technique.

### Establishing epistemic trust and a therapeutic alliance in the context of parents' ACEs

As noted above, childhood trauma is often associated with epistemic mistrust, given that the child did not experience the hoped for understanding and protection from the attachment figure (25). As parents, these ACEs may lead them to be hypervigilant and suspicious of the need for therapy or its relevance and effectiveness for the child and themselves. Mentalization theory suggests means to moderate this epistemic hypervigilance. Translating these means into therapeutic work with parents includes exploring the parents' expectations of the treatment openly and agreeing on relevant treatment goals and ways to achieve them (37). Additional means include mutual collaborative discussion of the child and the parents’ needs, believing in the parents' good intentions (22, 72) and using reasoned flexibility (60) when agreeing on the treatment setting for the parents’ meetings. The therapist's mentalizing of the parent's state of mind and defensive strategies in light of the parent's traumatic history can lead to a genuine acceptance of the parent's way of viewing things and willingness to join the parent where he or she is in terms of the different mentalizing poles. Moreover, meeting the parent where he or she is and then expanding rather than interpreting or refuting the parents' way of thinking can help overcome their defensive resistance, further reducing their hypervigilance and rigidity. Explaining mentalization theory in understandable terms and sharing developmental research findings regarding epistemic trust and tending to infants' needs as enhancing learning [e.g., (73)] is another way to help build the parents' trust in the process and the usefulness of mentalization techniques. Furthermore, the mentalizing-oriented therapist recognizes the separateness of minds and therefore takes responsibility for misunderstandings and repairing them (74), which can also increase the parent's trust in the process. Parents’ suspicions about the therapy, dismissal of its effectiveness and lack of trust in the therapist's good intentions may undermine the therapist's own mentalizing stance. Therefore, therapists must attend to and reflect on their own mental states, in-the-moment or in reflective supervision, particularly with regard to their arousal and anger.

*A vignette*

Mrs. B referred her 12-year-old daughter to therapy because of the child's suspected eating disorder. When asked during the intake about her own childhood, Mrs. B described growing up with an abusive, violent, punitive mother. The therapist acknowledged Mrs. B's painful history and then, understanding the importance of identifying angels in the context of a parent's traumatic history (75), asked Mrs. B about benevolent experiences during her childhood. Mrs. B responded to the question with intense fury, which puzzled the therapist, who felt under attack. This led to a momentary loss of the therapist's mentalizing stance. Noticing this response within herself *[therapist's self-reflection],* the therapist worked to regulate herself by “taking a deep breath”*,* and then asked how Mrs. B experienced the question *[re-establishing her therapeutic mentalizing, inquisitive stance, identifying Mrs. B's hypervigilance and asking about it]*. Mrs. B replied that the question implied that she was focusing only on the bad things in her past. The therapist apologized for her failure to fully appreciate Mrs. B's state of mind and her seemingly insensitive query *[recognizing the separateness of minds; taking responsibility for misreading the mother's internal state]*. The session was now back on track and they moved back to focusing on Mrs. B's concerns regarding her daughter *[joining the parent where she is and respecting her way of thinking]*. Only months later did Mrs. B identify her aunt as her childhood angel, concluding that her childhood loneliness and anger overshadowed and interfered with her ability to register any positive experiences *[connecting past with present experiences]*.

### Regulating the parents' emotional arousal

Scholars agree that acknowledging the impact of trauma on parenting may help parents build a coherent understanding of their parenting challenges (8, 12). Linking current difficulties with past relational histories also frees parents from their rigid defensive maneuvers that are needed to keep unconscious mourning and fantasies at bay (76). However, as noted above, parents may not see the relevance or cooperate in discussing their painful histories when pressured by challenges regarding their children's troubling behaviors. Furthermore, the discussion of the trauma and its consequences may bring up intense negative feelings such as shame, guilt, helplessness and pessimism, which increase stress and impede the parents’ ability to mentalize effectively. Furthermore, the accompanying emotional pain may make the sessions unbearable for the parent, leading to withdrawal from and/or disengagement in the therapeutic process. Therefore, the therapist has to monitor the parents’ arousal when discussing their childhood history and use downregulation or upregulation strategies as needed.

Building on developmental observations and research regarding the use of marked affect mirroring, slow-down talking, contingent communication and ostensive cuing (e.g., direct eye contact, head tilting, a soft tone of voice, leaning forward), mentalization theory demonstrates how parents help their children mentalize their experiences (43, 77). Similarly, mentalization-based interventions use these techniques when parents have become dysregulated and have difficulty mentalizing. This is done by joining with the parent, slowing down the emotional talk and inviting the parent to mentalize-in-the-moment (25, 43). In addition, the non-judgmental acceptance of the full range of the parents' emotions and perceptions, and validating and normalizing their current emotions and reactions in light of their traumatic history help regulate intense emotions. Additionally, putting one's self in the parents' shoes as a child and voicing their unspoken emotions also helps reduce the parents' arousal and allows them to return to or build their own mentalizing stance. Finally, the discussion of links between the parent's past traumatic experiences and current parenting challenges is framed as a tentative hypothesis for the parent to consider rather than interpretations of unconscious material, increasing the parent's sense of agency (24, 43, 58).

*A vignette*

Mr. and Mrs. O referred their daughter, Danielle, 8-years-old, for psychotherapy because of her intense separation anxiety and clinginess to her mother. During the intake the parents were asked about their upbringing, explaining that past experiences may shape current parenting *[therapist being explicit about the reason behind the question to reduce epistemic vigilance]*. Mrs. O reported growing up in a divorced family with a great deal of conflict, where she was separated from her father without an explanation. She talked reflectively about the pain of missing her father and longing to meet him, as well as the complicated, painful circumstances that led to her parents' divorce *[indicating a non-defensive, coherent account of past experiences]*. As the treatment progressed, Mrs. O recognized her tendency to be overprotective of Danielle, linking it painfully with her own childhood history *[self-reflection regarding her parenting and linking it coherently to her childhood trauma]*. However, following this reflection, Mrs. O became very distressed and guilt-ridden, assuming complete responsibility for her child's difficulties and expressing a very pessimistic view of her future.

Noting the mother's intense arousal, the therapist used ostensive cues to validate her worries and concerns, coupled with slowing down her emotional talk and mentalizing her emotions with words *[joining her emotionally, validating her experience and down-regulating arousal]*. The therapist also praised the mother's courageous introspection regarding her parenting. The willingness to see things from the mother's perspective, the non-judgmental approach and the slowing down led to a reduction in the mother's arousal. This paved the way for moving on from the mother's self-focused, critical perspective to a wider perspective, which included the mother's positive intentions and parental sensitivity *[shifting from the exclusive focus on her own painful emotions to an expanded, more balanced, self-compassionate perspective]*. The therapist also remarked on the girl's slow-to-warm up temperament, suggesting that it might have also contributed to Mrs. O's protective parenting *[shifting from self-focus to child-focus and a focus on the relationship].* Only when Mrs. O's arousal abated did the discussion move to alternative ways of responding to Danielle's clinginess and anxiety.

### Importance of following the parents' lead in the context of ACEs

When working to enhance parents' mentalizing in general, and in the context of ACEs in particular, it is advisable that the therapist follow the parents' lead and pace. In line with mentalization theory and techniques, the therapist is advised to be curious about and open to the parents' understanding of their child's problems before trying to intervene. When it comes to parents who have endured ACEs, it is tempting to interpret the parents' perceptions and reactions to the child in light of the parents' childhood trauma, which may indeed be the case. However, mentalization theory holds that it is the parents' willingness to explore and think reflectively and flexibly about their behavior and that of their child, that needs to be encouraged. In line with this goal, the therapist tries to let the parents' mentalizing abilities evolve at their own pace. Only when the parent expresses curiosity regarding the source of his or her attitudes and behavior should the therapist raise the possibility of the childhood trauma playing a role. This approach can also help parents traumatized in childhood who are prone to feeling helpless and passive feel more competent and capable (26). It also offers parents a model of how to use their mentalizing skills with their child and follow the child's lead.

*A vignette*

When Mr. O, the father of Danielle from the above vignette, was asked about his childhood, he reported that his father was a veteran who suffered from PTSD. He described the father as shifting between angry outbursts, withdrawal and dissociation. When asked what it was like for him to grow up in such circumstances, he replied dismissively and with blunt affect that these mood swings were expected given his father's PTSD diagnosis and elaborated no more. During one of the parents' sessions, the therapist tentatively suggested that Mr. O's insistence on Danielle becoming self-reliant had to do with the father's ACE of growing up with an unpredictable father. Mr. O responded with anger, saying that this had nothing to do with his daughter's symptoms and that he expected the therapist to focus on practical solutions rather than analyzing him. The therapist, shaken by the father's criticism and anger, had to work to regain her mentalizing stance *[self-reflect on her own emotions, link them with the stressed interaction and reestablish her inquisitive, empathic stance towards the father's inner experiences]*. The therapist did so by her reminding herself of Mr. O's painful history and his coping strategies as well as his parental devotion to his daughter. She then responded to Mr. O's comment by taking responsibility for suggesting an explanation that appeared irrelevant to him, indicating that her mind worked differently than his *[taking responsibility for the rupture and highlighting the separateness of minds]*. Following this intervention, the father's anger abated. In line with the collaborative approach advocated here, the therapist also joined Mr. O's wish to focus on Danielle's behavior as the chosen “port of entry” (78) for the parental meetings. This approach was useful in enlisting the father's involvement and cooperation with the parental psychotherapy. Only later, when more epistemic trust was created, did the therapist encourage Mr. O to seek his own psychotherapy, which he did after his daughter's symptoms improved.

### Handling parents' non-mentalizing modes in the context of ACEs

As noted above, some parents who have endured ACEs develop fragile mentalizing skills that are susceptible to collapse, especially in the face of reminders of their traumas. Under such circumstances, parents may collapse into non-mentalizing thinking, as expressed by the teleological, pretend or psychic equivalent modes. The mentalization-based intervention guidelines provide helpful strategies to handle these situations. Accordingly, the therapist is advised to stop the parents' dialogue empathically, yet firmly, and try to restore reflective, balanced, integrative and coherent thinking (55, 79). One method of doing so is having the therapist reflect on his/her difficulty following the parent's reasoning. The therapist can also ask the parent to slow down and rewind to an earlier point where mentalizing was intact. At other times the therapist may share his/her observation regarding the emotional intensity of the parent's speech and ask out of curiosity what brought it up and how it is linked with the shift in thinking. If the parent appears fixated on a certain pole or dimension of mentalizing, the therapist is advised to use empathy, validation and joining, indicating his/her willingness to view things from the parent's perspective and then try to shift, expand and widen the parent's perspective. This is done in conjunction with pacing the discussion and regulating the parent's arousal to a more optimized range, as discussed above.

A traumatized parent with the tendency to collapse into a **teleological non-mentalizing mode** will focus only on the circumstantial aspects of the child's behavior and the parent-child relationship, and be unwilling to consider the role of the child's or the parent's mental states in these occurrences. While in this mode, the parent is likely to seek a quick fix of the behavioral problem and may try to enforce his/her parental authority to achieve a change (22). When in this mode, the parent may resist talking about the trauma, which is regarded as an irrelevant diversion from the search for a solution to the child's behavior.

*A vignette*

Mr. O, Danielle's father, asked the therapist insistently for behavioral guidance on how to stop Danielle's anxiety and dependence on her mother *[a search for a behavioral solution]*. The therapist explained her perspective that for a solution to be relevant and work, they needed to explore the underlying motives behind the interactions between Danielle and her parents *[explicit discussion of the treatment strategy and the importance of mentalizing]*. She added that because Danielle was not present during the discussions, her thoughts, feelings and motivations could only be speculated on *[modeling the tentativeness and inferential nature of mental states]*. She suggested that a good starting point would be if the parents could reflect on their own mental states when faced with Danielle's anxious behaviors. Mr. O responded with assertion and anger that Danielle's behaviors were the product of Mrs. O's overprotective parenting and pampering *[an “all-knowing”, accusatory, non-mentalizing stance]*. Then his demeanor changed and he went on to state his strong belief that children can become self-reliant and independent only if they are left to cope on their own *[moving from the emotional and fixating on the cognitive polarity].* Mrs. O responded by accusing Mr. O of terrorizing and traumatizing Danielle *[the two escalating into a polarized non-mentalizing cycle]*. The therapist stopped the discussion, saying that the parents' mutual criticism and disagreements appeared to be leading to a dead end *[stopping the cycle of non-mentalizing]*. Then, in line with their agreed-upon decision to focus on Danielle's behavior as the chosen port of entry, she introduced the circle of security visual demonstration [COS, (80)] to the parents. She explained about attachment theory's claim of children's need for emotional support and regulation in order to promote their exploration and independence *[providing information on which to build parental mentalization]*. She then asked the parents if they found this information relevant for their daughter's behavior *[fostering a collaborative discussion]*. Mr. O considered the new information and concluded that it did help him understand his daughter's otherwise incomprehensible dependence. In the next few sessions Mr. O demonstrated how this new understanding helped him gradually notice new behaviors and emotions related to Danielle's slowly emerging self-reliance and independence *[fostering reflective observation, which serves as the building blocks for parental mentalization]*. These revelations, in return, opened the father up to acknowledging his own anxieties regarding Danielle's future and how they affected his parenting, thus showing better parental mentalization.

**The pretend non-mentalizing** mode is characterized by the extensive use of pseudo-psychological and intellectual reasoning. It is thought to be a defense of parents who endured ACEs against the pain of reliving traumatic events. Parents who use or collapse into this non-mentalizing mode will talk about their trauma with what sounds like reflection. However, the reflection will be mostly intellectual, self- and inner-focused and dissociated from external reality and emotional connectedness. They will acknowledge the trauma, but its recounting will sound remote and dissociated. When in this mode, the parents are likely to have a hard time recognizing their child's mental needs and/or fail to take action to ease the child's distress in a helpful way (22, 43).

*A vignette*

Mr. and Mrs. M wanted a second opinion regarding their 5-year-old son's recent diagnosis of Autism Spectrum Disorder (ASD). When asked about psychopathology in their families, Mr. M said nonchalantly that his brother was diagnosed with ASD as a child. As a result, the grandparents became totally invested in dealing with his brother and ignoring Mr. M's needs. Then he went on to dismiss the diagnosis given to his son, being very critical of the assessment process and the recommendations given to them at a child developmental and rehabilitation center. Given their disappointment with the system, the parents were planning to set up an ambitious, home-based treatment plan for their child, about which they talked in great detail.

The therapist, listening to the parents' talk, noticed the incoherence and contradiction in the parents' refutation of their son's diagnosis on one hand, and the certainty that they expressed over the home-based, ASD-based treatment plan *[typical of the pretend mode]*. Her sense was that the parents were fleeing into fantastic plans because they were flooded with anxiety. However, none of that anxiety was evident in the consultation room *[the lack of emotions, the hyper speech and the fantastic planning were understood as representing the non-mentalizing pretend mode]*. She thought that she had to stop the non-mentalizing talk but was worried that the parents might regard this move as criticism of them. Using her mentalizing stance she was able to authentically join the parents in their hope to provide their child with the best possible comprehensive plan. She then shared that if she were “in their shoes” she might have found it difficult to hear that their child might have the same diagnosis as the uncle. However, these were her thoughts and she was curious to know what it was like for them *[modeling her own self-reflection and expressing curiosity regarding the parents' inner experiences]*. Following her invitation, Mr. M became silent and after a while, she noticed tears in his eyes. At this point the father openly shared his pain over his son's diagnosis. The atmosphere in the room became sad and the therapist acknowledged the pain and fear that the parents were experiencing. After making space for these feelings, the therapist shifted the focus from the parents to the child, asking about his functioning and skills. At this point the parents were able to acknowledge their child's strengths along with his developmental difficulties, presenting a more balanced view of his needs.

At other times traumatized parents may all too rapidly and globally link their ACEs to whatever happens between them and their children. If this attribution is reparative, rigid and done without attention to nuances or the willingness to think about alternatives, it may reflect the **psychic equivalence non-mentalizing mode**. Traumatized parents in this mode may see potential trauma everywhere and have difficulty separating their past traumatic experiences from their current realities and those of their child (22). They may also fail to appreciate the separateness of minds, therefore failing to recognize that how they see events, which is overshadowed by their traumatic experiences, may not be how their child, spouse or the therapist sees them.

*A vignette*

Ms. A, a single mother, and her 7-year old son, were referred for child therapy and accompanying parent consultation because of the boy's very disruptive behavior at school. When asked about her own childhood, Ms. A shared that she grew up in a kibbutz where child communal sleeping was practiced. She recalled painful memories of having nightmares and not having her parents around to soothe her. She said with a mixture of anger and anguish that the communal upbringing ruined her life. Consequently, she made every effort to avoid unnecessary frustrations and consult the child on every decision related to him. Ms. A confided that whenever her son protested or cried, she was flooded with worries that she was hurting and traumatizing him. She recognized that her permissive parenting was extreme and may have contributed to her son's behavior problems at school *[which may reflect a mentalizing stance]*. However, she felt that reprimanding her son or not fulfilling his wishes would be unbearable and traumatic for him *[experiencing her anxiety of hurting her son as actual reality and confusing her own experiences and those of the boy].* The therapist felt that it was important to explain to the mother the risks associated with too permissive parenting *[an effort to move from an emotional and mental state focus to cognitive reasoning and a focus on external reality].* In response, the mother said with fury that only those who had experienced harsh caregiving know what it is like *[certainty and finality about reality]* and accused the therapist of trying to force her to adopt old-fashioned parenting practices *[hypervigilance and sense of persecution]*.

While this was happening, the therapist realized her own collapse into a judgmental, didactic, non-mentalizing mode, which may have had to do with the mother's anger at her *[self-reflection on the part of the therapist and re-establishment of a mentalizing stance]*. To repair the rupture, the therapist chose to validate the mother's saying that nobody could understand her past and present anguish and apologizing for her intervention, which the mother was correct in regarding as patronizing. Then, the therapist turned to discussing with Ms. A what it was like *for her* when her son responded with distress to the mother's demands *[conveying interest in her experiences, which is important in establishing epistemic trust; exploring the mother's own emotions with her before moving to the child]*. The therapist normalized the difficulty, common among parents who have endured ACEs, of separating between past and present and between the mother's childhood circumstances and those of her son *[building a coherent narrative of the parenting challenge and how it led to the psychic equivalence mode].* She also noted that it might be hard at times to differentiate accurately between the gradual, developmentally appropriate challenging of her child vs. traumatizing the child *[thinking in extremes, black and white thinking, lack of attention to nuances, which characterize the psychic equivalent mode]*. Later, the mother shared that this discussion helped her understand her motives in a more rational way and engage in more balanced parenting practices.

## Discussion and conclusions

In this paper we argued and demonstrated the usefulness of mentalization theory in conceptualizing the obstacles some parents who have endured ACEs encounter when dealing with parenting challenges related to their children's mental health and behavioral difficulties. We reviewed the empirical and clinical literature linking ACEs with the formation of fragile parental mentalizing skills, which make parents more susceptible to dysregulation and repeated mentalizing collapses when faced with parenting challenges. As a result, they fail to understand and support their children's emotional needs. Given the literature documenting the effectiveness of interventions that enhance parental mentalization and reflective functioning with parents of infants and toddlers, mostly mothers, we maintained that this approach can be useful with parents of older children as well. Specifically, we argued that offering the parents an intervention that promotes their mentalizing, whether stand-alone psychotherapy for the parents or in conjunction with their child's psychotherapy, can be helpful in several ways.

First, mentalizing the parents' needs in light of their adverse childhood experiences and current challenges can help them build a coherent narrative of their parenting challenges and ease their shame and guilt. Second, when parents' mentalizing skills become more stable, they may be more capable of providing consistent, sensitive parenting to the child in need. Third, developing a parental mentalizing stance can also help a parent who was the victim of childhood trauma become aware of situations of emotional dysregulation in the context of the parent-child relationship and find ways to self-regulate. Moreover, the collaboration that characterizes the mentalization-based clinical approach can empower parents and increase their resilience and competence, all important factors in mitigating the risks associated with childhood adversity. Finally, the centrality of the therapist's mentalizing stance, a major tenant of mentalization techniques, is especially important when working with parents who endured ACEs. As our clinical examples demonstrated, the therapist's self-reflection and mentalizing stance helped in identifying instances of emotional dysregulation in the therapist and in the parent. These techniques also helped them recognize events of hypervigilance and therapeutic ruptures. As a result, moments-of-meetings (78) occurred through which the parents and the therapist co-created a mutually lived story between them.

To the best of our knowledge, tying together the research on parenting, ACES and parental mentalization, and discussing the benefits of mentalization-based techniques as treatment guidelines for parents who endured childhood trauma has not been done before. When addressing a parent's ACE in the context of parent psychotherapy, we recommend keeping in mind that when parents bring their child for psychotherapy, they are not necessarily open and/or willing to work through their own relational histories. To foster epistemic trust and build parental mentalization skills, the therapist may share what is known about the impact of ACEs on parenting and then follow the parent's lead on how much to explore the past. In line with the mentalization theory perspective, focusing attention on the parents' traumatic history is warranted when they express curiosity about the possible reasons behind their behaviors and mental states, and when linking them to the trauma appears to provide a plausible, coherent narrative for them. In this context it is important to recall that mentalization-based therapy is primarily process-focused rather than content-focused (43). Thus, the therapeutic work can focus less on the details of the parents' traumatic history, especially if they oppose this focus (as was the case with Mr. O). Rather, the work can focus on recognizing how the trauma may trigger strong emotions, or create “bumpy roads” (58) or “hot spots” (60) that may lead to mentalizing collapses and “vicious cycles of nonmentalizing” (22) between the parent and child. Such an awareness can promote self-regulation in the parent, as well as compassion and empathy in the spouse, which can facilitate co-parenting cooperation (81). The therapist can also help the traumatized parent find ways to repair mentalizing ruptures, normalizing their occurrence and contextualizing them in terms of parental stress and/or childhood ACEs.

It is important to keep in mind that discussing a parent's trauma, besides being dysregulating to the parent, may also be dysregulating for the therapist. Such discussions may activate the therapist's own painful childhood memories and increase the risk of the therapist's mentalizing collapse (82). Thus, reflective supervision (83), where the therapist is encouraged to raise therapeutic issues in a safe, non-judgmental environment and reflect on his/her own thoughts, feelings, experiences and mentalizing collapses and recovery, is highly recommended when working with parents who have endured ACEs. Just as the parent needs to be sufficiently regulated in order to regulate the child, the therapist has to be sufficiently aware and regulated in order to regulate and help the parent process his or her past trauma and how it is linked with parenting and the child's current difficulties.
