# Erratum: Therapeutic work to enhance parental mentalizing for parents with ACEs to support their children's mental health: a theoretical and clinical review

**DOI:** 10.3389/frcha.2023.1286714

**Published:** 2023-09-11

**Authors:** Frontiers Production Office

**Affiliations:** Frontiers Media SA, Lausanne, Switzerland

**Keywords:** parental mentalization, ACES, parent psychotherapy, parental reflective functioning, intergenerational trauma

An Erratum on Therapeutic work to enhance parental mentalizing for parents with ACEs to support their children’s mental health: a theoretical and clinical review By Dollberg DG and Hanetz-Gamliel K. (2023) Front. Child Adolesc. Psychiatry 2:1094206. doi: 10.3389/frcha.2023.1094206

Due to a production error, the conversion of the in-text citations led to mistakes throughout the body of the text that did not reflect the accepted manuscript.

The publisher apologizes for this mistake.

The original version of this article has been updated.

